# Endophytic fungi: nature’s solution for antimicrobial resistance and sustainable agriculture

**DOI:** 10.3389/fmicb.2024.1461504

**Published:** 2024-12-12

**Authors:** Asiya Nazir, Abdul R. Puthuveettil, Fathima Hasnain Nadeem Hussain, Khalid E. Hamed, Nayla Munawar

**Affiliations:** ^1^College of Engineering, College of Health Sciences, Abu Dhabi University, Abu Dhabi, United Arab Emirates; ^2^Department of Plant Protection, College of Agriculture and Food, Qassim University, Buraydah, Saudi Arabia; ^3^College of Engineering, Abu Dhabi University, Abu Dhabi, United Arab Emirates

**Keywords:** antibacterial compound, drug resistance, endophytic fungi, gene activation, natural product regulation

## Abstract

The growing threat of antimicrobial resistance (AMR) has underlined the need for a sustained supply of novel antimicrobial agents. Endophyte microorganism that reside within plant tissues as symbionts have been the source of potential antimicrobial substances. However, many novel and potent antimicrobials are yet to be discovered from these endophytes. The present study investigates the potential of endophytic fungi as a source of novel bioactive chemicals with antibacterial capabilities. These fungi synthesize secondary metabolites such as polyketides and peptides via polyketide synthase (PKS) and nonribosomal peptide synthetase (NRPS) pathways. Notable substances, like prenylated indole alkaloids and fumaric acid, have shown promising antibacterial and antifungal properties against multidrug-resistant infectious agents. This review also emphasizes the symbiotic link between endophytes and their host plants, which is critical for secondary metabolite production. The study focuses on the significance of isolation methods for endophytes and proposes their use in for sustainable agriculture, bioremediation, and medicine. Future research combining endophytic biodiversity analysis with next-generation sequencing (NGS) and nanotechnology could provide novel techniques for combating AMR and contributing to sustainability across multiple industries.

## Introduction

Endophytes are microorganisms that reside within plant tissues without causing harm and have garnered significant attention for their potential to produce antimicrobial substances (Jha, 2023). These microorganisms are found in a diverse range of plants, including trees, bushes, herbs, and grasses. Recent research has highlighted their ability to create compounds with high therapeutic potential, offering a sustainable and environmentally friendly alternative to traditional sources of medicinal agents (Sorrenti et al., 2023). Endophytes engage in complex interactions with their host plants, such as parasitism, mutualism, and antagonism, which can produce valuable secondary metabolites (Agri et al., 2022). For instance, marigold endophytes produce hydrolytic enzymes and Indole-3-acetic acid (IAA) like their hosts, showcasing their role in promoting host growth and stress resistance (Shurigin et al., 2021). This intricate relationship triggers a series of biochemical processes in the host, enhancing its ability to produce secondary metabolites. The sustainable cultivation and ease of isolation of endophytes make them a promising field of study for developing new drugs and treatments for infectious diseases. In contrast, the overharvesting of medicinal plants and ecological challenges have hindered the traditional approach of sourcing bioactive compounds from plants themselves (Palanichamy et al., 2019). As antimicrobial resistance becomes a significant public health concern due to rising multidrug resistance and diminishing efficacy of conventional antibiotics, the exploration of endophytes as a source of new antimicrobial agents is crucial (Salam et al., 2023). Endophytes offer a viable solution to enhance the effectiveness of treatments for infectious diseases and address the urgent need for new antimicrobial compounds. Reports of secondary infections like *Acinetobacter baumannii* and other microorganisms in patients with SARSCoV2 during the COVID-19 pandemic have coincided with the expansion of *A. baumannii’s* medication resistance in hospitals and healthcare settings due to frequent use of medical facilities and instruments (Kyriakidis et al., 2021). The COVID-19 pandemic has highlighted the critical need for new antimicrobial compounds to combat rising infectious diseases, thus emphasizing the relevance of endophyte research in the modern day. Endophytes present a practical way to improve the efficacy of treatments for infectious disorders and meet the pressing demand for novel antibacterial substances.

The production of bioactive metabolites by endophytes is inextricably linked to their vivid roles. According to recent studies, when endophytes promote the production of host secondary metabolites, their hosts do more than increase substances; they trigger a series of biochemical processes in their hosts, such as growth and stress resistance. Endophytes follow, two main methods to promote their host. They produce secondary metabolites like their hosts through the same signal pathway during gene mutation or information exchange. Marigold endophytes, for instance, produce hydrolytic enzymes and IAA like their hosts (Shurigin et al., 2021). Endophytes also work together with their hosts to complete the same signal pathway, producing key enzymes, or altering the direction of the host metabolism to produce specific metabolites (Trivedi et al., 2020). In addition to enhancing endophytes’ potential for therapeutic use, this dynamic interplay between endophytes and their host plants creates new opportunities for environmentally friendly farming methods. Endophytes have the potential to completely transform biotechnology, agriculture, and medicine as more research reveals the intricate mechanisms underpinning these connections.

## Endophytes and their isolation

Endophytes include both bacteria and fungi, which can be classified as bacterial or fungal endophytes, each possessing unique roles and abilities. They coexist symbiotically with the plant, frequently providing their hist with various advantages in exchange for nutrients and a protective habitat. Endophytes inhabit plant tissues’ intercellular or intracellular spaces, including roots, stems, leaves, and seeds (Afzal et al., 2019). Their ability to produce a wide range of bioactive compounds and enhance plant resilience to various biotic and abiotic stresses makes them valuable allies in addressing global challenges related to food security, degradation of environmental pollutants, and the development of new pharmaceuticals (Leichtweis et al., 2021). They provide plants with several benefits, including improved growth and disease resistance. Several other applications for endophytes have already been explored in other fields, including agriculture, bioremediation, and pharmaceuticals (Arora et al., 2022). Endophytic microorganisms, though not fully studied, are recognized as valuable resources in various research.

**Figure 1:**
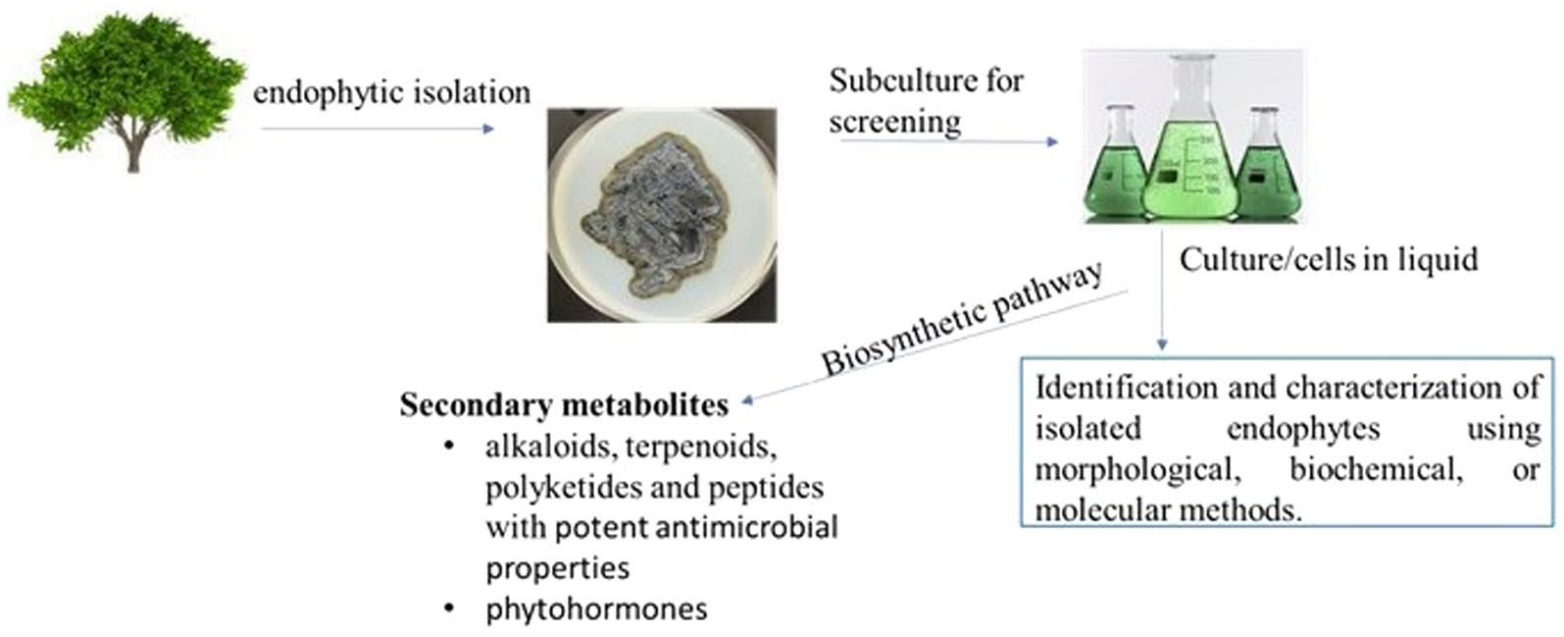
Process of Isolating Endophytes from Tree Samples: Surface Sterilization, Plating, Incubation, and Screening of Secondary Metabolites.

Isolation of endophytes involves the surface sterilization of host plant tissues using 2–10% (v/v) sodium hypochlorite (NaOCl) Ethanol (70–95%), serves as the preferred wetting agent due to its minimal antibiotic effect. After sterilization, samples are rinsed with sterile water or 70–95% ethanol to remove any residual sterilant. Generally, sterilized plant segments are plated on malt extract agar, potato dextrose agar, and nutrient agar, which are commonly used for isolation of fungi and bacteria, respectively. For primary isolation, colony-limiting agents and antibiotics are often used. The isolated pure colonies of either fungus or bacteria are then further characterized and taxonomized. Figure 1 illustrates the process of isolating endophytes from tree samples, starting with surface sterilization, plating on nutrient agar, and incubation and screening of secondary metabolites.

### Antimicrobial properties and biosynthetic pathways of antimicrobial compounds in endophytes

Antibiotics, antivirals, antifungals, and antiparasitic are examples of antimicrobials, which are drugs used to treat and prevent infectious diseases in humans, animals, and plants. Antimicrobial resistance (AMR) is a critical global health issue where microorganisms evolve to resist the effects of antimicrobial agents, rendering standard treatments ineffective and leading to persistent infections and increased mortality rates (World Health Organization, 2014). Due to the growing threat posed by drug resistant microbes, it has become crucial to find innovative antimicrobials to combat antimicrobial resistance. Endophytic fungi produce a variety of bioactive compounds, including alkaloids, terpenoids, polyketides, and peptides, which exhibit potent antimicrobial properties. *Cryptosporiopsis quercina*, an endophyte was reported from *Tripterigeum wilfordii*, a medicinal plant native to *Eurasia* which demonstrated excellent antifungal activity against some important human fungal pathogens—*Candida albicans* and *Trichobn* species. This compound contains several peculiar hydroxylated amino acids and a novel amino acid: 3hydroxy4hydroxy methyl proline (Strobel et al., 1999). Lin et al. (2021) isolated three prenylated indole alkaloids from the endolichenic fungus *Aspergillus chevalieri*, namely Asperglaucins A and B (1 and 2), and Neoechinulin F. The researchers found that all compounds had potent antibacterial activities against *Pseudomonas syringae* pv actinidae (Psa) and *Bacillus cereus*. El-Zawawy et al. (2023), originally reported the presence of L-tyrosine in *Rhizopus oryzae*, which was isolated from *Opuntia ficus-indica* L. Strong antibacterial and antibiofilm activities were demonstrated by this compound against multidrug-resistant bacterial strains (MIC values ranging from 6 to 20 μg/mL) isolated from burn wound infections. These strains included *P. aeruginosa* PA02, PA09, *E. coli* EC03, *Klebsiella pneumonia* KP01, *S. aureus* SA03, and *S. aureus* SA04. According to Nazir et al. (2022), chromatography revealed the production of fumaric acid and chrysophanol, two more secondary metabolites by *Fusarium redolens* which was isolated from *Olea europaea*. Endophytic bacterium *Aeromicrobium ponti*, isolated from the medicinal plant *V. divergens* yielded six chemicals namely 1acetyl-*β*-carboline, indole-3-carbaldehyde, tryptophol, 3(hydroxymethyl) indole, brevianamide F, and cyclo (L-Pro-L-Phe) which showed moderate antibacterial activity against methicillin-sensitive and methicillin-resistant *S. aureus* with inhibition zones measuring 10–18 mm and 8–15 mm, respectively (Gos et al., 2017).

Endophytic fungi employ several biosynthetic pathways to produce antimicrobial compounds, with the polyketide synthase (PKS) and nonribosomal peptide synthetase (NRPS) pathways being particularly significant. Polyketide synthases (PKS) are large, multifunctional enzymes responsible for synthesizing a variety of secondary metabolites, including antibiotics, antifungals, and anticancer agents. These enzymes catalyze the assembly of complex polyketides from simple acyl-CoA precursors through a series of condensation reactions. For instance, Sheridan et al., (2015) reported that the endophytic fungi and bacteria containing PKS genes produce epipolythiodioxopiperazines (ETPs), cyclic peptides, and polyketides, which have notable antimicrobial properties. Similarly, non-ribosomal peptide synthetases (NRPS) are modular enzymes that synthesize non-ribosomal peptides, which are crucial for various biological activities, including antimicrobial action. NRPSs function by sequentially adding amino acids to a growing peptide chain without the direct involvement of ribosomal machinery. These enzymes play a pivotal role in the biosynthesis of peptide antibiotics, such as those produced by endophytic microorganisms (Muna and Josphat, 2014).

In addition to PKS and NRPS pathways, other genetic mechanisms contribute to the production of antimicrobial compounds in endophytes. For example, endophytes can produce compounds like camptothecin and taxol, which have significant pharmaceutical applications (Xie et al., 2020). The integration of these pathways and the compounds they produce underscores the potential of endophytic fungi in generating diverse and potent antimicrobial agents. By utilizing these biosynthetic pathways, endophytes offer a sustainable and innovative approach to developing new antimicrobial drugs, addressing the urgent need for novel treatments in the face of rising antimicrobial resistance (Nazir et al., 2022; Shurigin et al., 2021). Terpene cyclases utilize the mevalonate pathway to produce terpenoids, which are made up of multiple isoprene units. This process encompasses the synthesis of carotenoids through phytoene synthases, indole diterpenes via prenyltransferases, and cyclic terpenes formed by diterpene cyclases and sesquiterpene cyclases (Zeilinger et al., 2016). On the other hand, the shikimic pathway is used to produce indole alkaloids, polyketides are made from acetyl-CoA and malonyl-CoA units by polyketide synthases, and NRPS enzymes generate nonribosomal peptides without the need for ribosomes. Consequently, various biosynthesis pathways produce distinct fungal metabolites (Zeilinger et al., 2016). Devi et al. (2012) extracted penicillin and nonribosomal peptides (NRPs) from *Penicillium chrysogenum* MTCC 5108, an endophytic fungus found in mangroves. Similarly, a screening technique based on polyketide synthase I (PKS I) led to the discovery of a novel polyketide from endophytic fungi called *Penicitriketo* (Wang et al., 2014).

The genome of the fungus *Calcarisporium arbuscula*, an endophyte connected to the *Russulaceae* family, has reportedly been examined using antiSMASH, according to Cheng et al. (2020). A total of 65 biosynthetic gene clusters (BGCs) were found to oversee the production of secondary metabolites by this investigation. Particularly, genes encoding nonribosomal peptide synthetases (NRPSs) and polyketide synthases (PKSs) were discovered in 12 and 23 gene clusters, respectively. Furthermore, it is anticipated that a few gene clusters control the synthesis of several mycotoxins, such as aflatoxin, citrinin, aurovertins, alternariol, destruxins, and isoflavipucine.

Whole genome sequencing of *Pestalotiopsis fici*, an endophyte from the branches of the tea plant *Camellia sinensis*, demonstrated a wide range of metabolic capabilities that emphasizes its potential relevance in numerous biological processes in a study by Wang et al. (2014). A total of 27 polyketide synthases (PKSs), 12 nonribosomal peptide synthetases (NRPSs), 5 dimethylallyl tryptophan synthases, 4 putative PKS-like enzymes, 15 putative NRPS-like enzymes, 15 terpenoid synthases, 7 terpenoid cyclases, 7 fatty acid synthases, and 5 PKSNRPS hybrids were found by the researchers using antiSMASH analysis. There are 74 biosynthetic gene clusters (BGCs) that include the genes for these important enzymes. Cochlioquinone B, D, 8Hydroxy-6-methyl-9-oxo-9H-xanthene-1-carboxylic acid methyl ester, and isofusidienol A are the four chemicals that have been identified from *Helotiales* sp., an endophyte of the medicinal plant *Bergenia pacumbis*. Twenty-seven biosynthetic gene clusters (BGCs) encoding 26 type I PKSs, 3 type III PKSs, 25 NRPSs, 6 PKS/NRPS hybrids, 13 terpenes, and 4 indoles were found in the endophyte’s genome according to an antiSMASH analysis. Additionally, the production of four terpene genes was codon optimized for *Streptomyces* spp. Consequently, the recombinant strains were able to effectively synthesize an unidentified terpenoid and linalool, both of which are frequently found in plants, as well as their oxidized form (Tao et al., 2022).

### Application of endophytes

Endophytic fungi hold great promise in bioremediation, utilizing their enzymatic pathways to degrade environmental pollutants such as heavy metals and hydrocarbons (Guo et al., 2010; Wan et al., 2012). In sustainable agriculture, they enhance plant growth by improving nutrient uptake and producing phytohormones like auxins and gibberellic acid, promoting stress tolerance (Rashid et al., 2012; Nacoon et al., 2020) (Table 1).

**Table 1:** Applications of endophytic fungi.

Application	Description	Examples	References
Antimicrobial Agents	Endophytic fungi produce a range of secondary metabolites such as alkaloids, polyketides, and peptides that exhibit potent antimicrobial properties. These compounds are critical in combating antimicrobial resistance (AMR) by providing novel therapeutic agents for multidrug-resistant infections.	Prenylated indole alkaloids from *Aspergillus chevalieri*, showing strong antibacterial activity against *Pseudomonas syringae* and *Bacillus cereus*	Nazir et al. (2022) and Jha (2023)
		Fumaric acid produced by *Fusarium redolens*, with antimicrobial effects against various pathogens	
Biocontrol in agriculture	Endophytic fungi serve as natural biocontrol agents that inhibit plant pathogens. They help improve crop health by competing for nutrients, producing inhibitory compounds, and inducing plant defense mechanisms, which reduce the need for chemical pesticides.	*Trichoderma* species, producing hydrolytic enzymes and antibiosis against *Rhizoctonia solani* and *Botrytis cinerea*, improving plant resistance to diseases	Alsalamah et al. (2024)

### Role of endophytes in agriculture: biocontrol, stress tolerance, and plant growth promotion

Endophytes are useful in agriculture as biocontrol agents against pathogens that cause plant diseases. Endophytes produce compounds that inhibit harmful microorganisms’ growth while promoting beneficial ones’ growth. As a result, they are an eco-friendly alternative to chemical pesticides (Alsalamah et al., 2024). In addition, some endophytes can enhance the plant’s ability to cope with abiotic stresses, such as drought and salinity. Endophytes provide direct assistance to plants, either by creating phytohormones like auxins, IAA, gibberellic acid, and ethylene, or by helping plants absorb nutrients from the environment such as phosphorus, iron, and nitrogen (Muhammad et al., 2024; Paliwal et al., 2023). By inhibiting phytopathogens that cause diseases, endophytes can tangentially benefit plant growth. Similar, but different, mechanisms can be used by rhizospheric and endophytic bacteria and fungi to transfer effects to host plants that promote plant growth (Rashid et al., 2012). Some endophytic bacteria generate phosphatases, which help with the mineralization of organic phosphorus. It has been noted that the bacterial endophytes of *Burkholderia*, *Bacillus*, *Methylobacterium*, *Bacillus, Pantoea*, *Klebsiella*, and *Rhizobium* spp. function as phosphate solubilizers (Rana et al., 2020). Species of *Aspergillus, Penicillium,* and *Trichoderma* are also known for their biocontrol efficacy against fungal plant pathogens by producing antifungal compound and disintegration of DNA of pathogen (Khan and Javaid, 2020; Khan and Javaid, 2022a; Khan and Javaid, 2022b; Anbalagan et al., 2024; Dave et al., 2024; Vinothini et al., 2024; Zope et al., 2019). They function as important biocontrol agents in agriculture, reducing plant diseases through competition, parasitism, antibiosis, and inducing plant defensive responses (Wang et al., 2022). *Aspergillus* spp. inhibit infections by creating secondary metabolites like gliotoxin, and nontoxic strains are used to control toxigenic species, particularly in maize and peanuts. Some strains also produce enzymes that break down pathogen cell walls, hence reducing root infections. *Penicillium* spp. employ bioactive substances such as penicillin and patulin to combat soilborne diseases caused by *Fusarium* and *Pythium*, while also encouraging plant development by secreting auxins and increasing nutrient availability (Haq et al., 2024; Arora et al., 2022). *Trichoderma* spp. is among the most efficient biocontrol fungus, attacking diseases such as *Rhizoctonia solani* and *Botrytis cinerea* in which they adhere to and breakdown the infections’ hyphae. These fungi also release hydrolytic enzymes, such as chitinases and glucanases, which degrade fungal cell walls and promote systemic resistance in plants, therefore boosting their natural defenses. Collectively, these fungi serve an important role in sustainable agriculture by lowering the need for chemical pesticides, enhancing plant health, and improving resilience to diseases (Mehta et al., 2019; Tyśkiewicz et al., 2022). By saturating the phosphorus input into the plant root system, endophytic *Curvularia geniculate* from *Parthenium hysterophorus* roots have been shown (Priyadharsini and Muthukumar, 2017) to be capable of promoting plant development. According to Nacoon et al. (2020), phosphate-solubilizing bacteria and AM fungi work together synergistically to promote plant development in *Helianthus tuberosus* L. The endophytes release antioxidant metabolites including glutathione and ascorbate that reduce reactive oxygen species and increase salt tolerance in the host cells. By enhancing root length and density, supplying more nutrients to plants, inhibiting phytopathogens, and enhancing relative water content, osmotic adjustment, and antioxidant properties, these mechanisms collectively boost plant growth under abiotic and biotic stress conditions (Choudhary et al., 2023). According to Etminani et al. (2022), grapevine endophytic bacteria, including *Pantoea* sp. Sa14, *Pseudomonas* sp. Sn48, *Pseudomonas* sp., *Pseudomonas* sp., *Serratia* sp., and *Enterobacter* sp., produce volatile organic compounds (VOCs) that hinder *Agrobacterium tumefaciens* growth in several ways, including by preventing motility, chemotaxis, root attachment, and biofilm formation (Verma et al., 2022).

### Role of endophytes in bioremediation: enhancing plant growth and pollutant degradation

In the presence of harmful heavy metals, endophytes can also promote plant growth, which has a favorable impact on the contaminant’s absorption, breakdown, and dislocation by the plants (Deng and Cao, 2017). Out of the endophytes isolated in his work, three species of *Penicillium* were identified. The potential of *Penicillium* species to biosorb and bioaccumulate harmful heavy metals has been well investigated in his research. Through intricate symbiotic relationships with their host plants, endophytes play a pivotal role in enhancing phytoremediation processes. Their unique metabolic pathways and enzymatic activities contribute to the degradation of pollutants, ranging from organic compounds to heavy metals, rendering them less harmful to the environment. Endophytes are known to employ a variety of enzymes to break down and transform pollutants into less harmful forms (Stępniewska and Kuźniar, 2013). For instance, in the degradation of organic pollutants, enzymes such as ligninases, peroxidases, and laccases play crucial roles by catalyzing the breakdown of complex organic molecules (Vasundhara et al., 2019). A study conducted by Guo et al. (2010) identified an endophytic bacterium, *Bacillus* sp. DNP, which, when combined with N, N′-dicyclohexylcarbodiimide (DCC), a specific ATPase inhibitor, was able to reduce cadmium levels by approximately 94%. Similarly, growth inhibition of *Solanum nigrum* L. in the presence of cadmium was lessened by adding *Serratia nematodiphila* LRE07 as an endophytic bacterium (Wan et al., 2012). Ma et al. (2015) isolated Ni-resistant endophytic bacteria from tissues of *Alyssum serpyllifolium* growing in serpentine soils. When this strain was injected into *Brassica juncea* seedlings, he observed a significant increase in plant biomass Gaber et al. (2023) mentioned *Phialocephala* spp. (TCDAs18R1B3) as dark septate endophyte, which was also later reported by Yamaji et al. (2016), having lower phytotoxicity and frequently observed in conjunction with plants growing in environments with high levels of heavy metals Chen et al. (2012) have also reported the use of endophytic bacteria L14, which were isolated from the *Cadmium hyperaccumulator* and *Solanum nigrum* L., in the bioremediation of heavy metals. Three strains from Cerrado plants that are capable of degrading various petroleum, diesel, and petrol components have been identified by De Oliveira et al. (2012). Growing in cadmium-contaminated soil and treated with an endophytic consortium, Bibi et al. (2018), proved that *Lactuca sativa* produced more biomass on the leaves and roots than the untreated group. It was also shown that the use of endophytes enhanced plant fitness under adverse circumstances and decreased cadmium buildup in the edible portion of lettuce, lowering the risk to human health (Enshasy et al., 2020). *S. griseus* was identified by Do et al. (2022) as viable options for bioremediation of copper ions from the contaminated environment. It has been demonstrated that metal concentrations affect the ability of bacteria to bind metals. To meet their metabolic needs, microorganisms are better at accumulating critical metals at low concentrations (Kamal et al., 2023; Sagar et al., 2020; Budamagunta et al., 2023; Naz et al., 2023).

## Limitations of using endophytic fungi-derived antimicrobials

While endophytic fungi provide a unique and sustainable supply of bioactive chemicals to prevent antimicrobial resistance (AMR), there are various limitations and potential adverse effects to consider (Table 2).

**Table 2:** Challenges and examples of endophytic fungi derived antimicrobials.

Limitation	Example	References
Toxicity	Hepatotoxic, nephrotoxic, neurotoxic effects; and gastrointestinal discomfort, dysbiosis.	Esheli et al. (2022) and Xing et al. (2022)
	Opportunistic infections caused by microbial imbalance due to potent broad-spectrum compounds.	Zhgun (2023)
Regulatory challenges	Safety assessment, toxicity research and trials and testing to find out the interaction with other medications must	Tiwari and Bae (2022)
Variability in metabolite production	Environmental factors (temperature, host plant type) cause inconsistency in bioactive compound yields.	Digra and Nonzom (2023) and Kumari et al. (2023)
	Strain-specific differences lead to unpredictable results in large-scale production.	Zeilinger et al. (2016)
Allergenic potential	Production of mycotoxins causing allergic reactions and chronic toxicity upon prolonged exposure.	Xing et al. (2022)
Ecological concerns	Overharvesting host plants and altering native fungal strains may disrupt ecosystems.	Hernandez and Martinez (2018)
	Potential resistance in non-target microorganisms.	Case et al. (2022)
Scalability and economic feasibility	Low yields of metabolites and complex extraction techniques requiring significant infrastructure investment.	Wen et al. (2022)
	Fungi may cease production after multiple culture cycles, further complicating scalability.	Singh and Kumar (2023)

### Toxicity

One of the most significant issues is toxicity and off-target consequences. Although certain fungal metabolites have significant antibacterial properties, they may also be cytotoxic to human cells or destroy healthy organs (Esheli et al., 2022). Secondary metabolites, such as alkaloids and polyketides, can be harmful in high quantities, resulting in hepatotoxicity, nephrotoxicity, and neurotoxicity. These chemicals, particularly those with broad-spectrum action, may also alter the delicate balance of beneficial microbiota in the human gut or on the skin, resulting in dysbiosis and other health problems such as gastrointestinal discomfort, diarrhea, or opportunistic infections (Zhgun, 2023).

### Regulatory challenges

They may hamper the transition of endophytic fungal metabolites from research to clinical usage. Regulatory organizations require substantial safety data before approving new antimicrobial drugs, and the unique nature of endophyte-derived compounds may necessitate extra toxicity research and clinical trials, adding time and cost. The possible interaction of these metabolites with other medications must also be thoroughly investigated to minimize undesirable drug interactions (Tiwari and Bae, 2022; Gupta et al., 2023).

### Variability in metabolite production

The variation in metabolite synthesis is another significant drawback. Endophytic fungi’s production of antimicrobial chemicals is frequently impacted by environmental variables such as temperature, humidity, the availability of nutrients, and the type of host plant. Because the same fungal strain may produce various amounts of bioactive chemicals under different conditions, this variability makes it more challenging to maintain the consistency needed for medication development (Digra and Nonzom, 2023). Another challenge is the strain-specific variations that complicate the assurance of consistent results on a large scale, particularly without sophisticated bioreactor systems for controlled cultivation (Kumari et al., 2023).

### Allergenic potential

A further issue is the allergenic potential of fungal metabolites, which may cause allergic responses or immunological hypersensitivity in sensitive persons. Some endophytic fungi create mycotoxins, which, although effective against infections, can cause chronic toxicity or allergic reactions with extended contact. To assure the safety of fungal-derived medicines, careful dosing and allergy screening will be required (Xing et al., 2022).

### Ecological concerns

When creating fungal-derived antimicrobials, it is also necessary to address environmental considerations. Overharvesting certain host plants or modifying native fungal strains for medication research may harm ecosystems by disrupting the plant-fungi relationship. This raises concerns regarding sustainability and environmental effects, especially if fungal strains are overexploited without proper conservation methods in place (Hernandez and Martinez, 2018). Furthermore, the introduction of endophyte-derived chemicals into agricultural or medicinal systems may cause unexpected effects, such as the development of resistance in nontarget microorganisms, thereby reducing the long-term efficacy of these therapies (Case et al., 2022).

### Scalability and economic feasibility

While isolating bioactive compounds from endophytic fungus is possible in a laboratory environment, generating these metabolites on a commercial scale is frequently difficult due to poor yields, complicated purification methods, and the requirement for specialized infrastructure (Wen et al., 2022). Fermentation and extraction procedures may require optimization to lower production costs, perhaps limiting their broad use in healthcare and agriculture. Furthermore, several fungi may stop producing essential metabolites after numerous culture cycles, hindering scaling (Singh and Kumar, 2023).

## Conclusion

Endophytes play a crucial role in plant defense by producing bioactive compounds, benefiting fields such as pharmaceuticals, agriculture, and environmental sciences. Advances in scientific techniques, especially next-generation sequencing (NGS), allow for detailed exploration of these symbiotic microbes, their metabolites, and the genes responsible for producing diverse bioactive agents. However, many endophytes remain undiscovered due to challenging cultural conditions. Further research into their ecology and bioprospecting efforts could unlock novel solutions for medicine, bioremediation, and sustainable agriculture. Through genetic engineering and nanotechnology, endophytes hold significant promise in addressing global challenges and promoting sustainability.
